# Supplementary material for: Kinetochores attached to microtubule-ends are stabilised by Astrin bound PP1 to ensure proper chromosome segregation
Source: eLife. 2019 Dec 6;8:e49325. doi: 10.7554/eLife.49325 (PMC6930079; doi:10.7554/eLife.49325)
Supplement: Supplementary file 1. [file elife-49325-supp1.docx]

**Supplementary File 1: Key resources table**

| **Reagent type (species) or resource** | **Designation** | **Source or reference** | **Identifiers** | **Additional information** |
| --- | --- | --- | --- | --- |
| Cell line (*H. sapiens*) | HeLa Flp-In | Thermo Fisher | R71407 | Kindly donated by Stephen Taylor |
| Cell line (*H. sapiens*) | HeLa Flp-In | This paper | YFP-Astrin WT siRNA res | Can be obtained from Draviam lab or Ximbio |
| Cell line (*H. sapiens*) | HeLa Flp-In | This paper | YFP-Astrin 4A siRNA res | Can be obtained from Draviam lab or Ximbio |
| Cell line (*H. sapiens*) | HeLa Flp-In | This paper | YFP-Astrin ∆70 siRNA res | Can be obtained from Draviam lab or Ximbio |
| Cell line (*H. sapiens*) | HeLa | [(Trinkle-Mulcahy et al. 2001)](https://paperpile.com/c/OldSzW/rvTE) | YFP-PP1_γ_ | Can be obtained from Draviam lab |
| Antibody | anti-α-Tubulin (rat monoclonal) | Abcam | ab6160 | IF (1:800 or 1:500) |
| Antibody | anti-γ-Tubulin (mouse monoclonal) | Sigma Aldrich | T5326 | WB (1:800) |
| Antibody | anti-GFP (mouse monoclonal) | Roche | 1181446001 | IF (1:800) |
| Antibody | anti-GFP (rabbit polyclonal) | Abcam | ab290 | IF (1:1000) |
| Antibody | anti-mCherry (rat monoclonal) | Thermo Fisher | M11217 | IF (1:2000) |
| Antibody | anti-mCherry (rabbit polyclonal) | Abcam | ab167453 | IF (1:2000) |
| Antibody | anti-SKAP (rabbit polyclonal) | Atlas | HPA042027 | IF (1:1000) |
| Antibody | anti-Astrin (rabbit polyclonal) | Novus Biologicals | NB100-74638 | IF (1:1000) |
| Antibody | anti-Astrin (rabbit polyclonal) | Proteintech | 14726-1-AP | WB (1: 3000) |
| Antibody | anti-SKA3 (mouse monoclonal) | Santa Cruz Biotechnology | sc-390326 | IF (1:500) |
| Antibody | anti-ZW10 (mouse monoclonal) | Abcam | ab53676 | IF (1:1000) |
| Antibody | anti-MAD2 (rabbit polyclonal) | Covance | PRB-452C | IF (1:500) |
| Antibody | anti-HEC1 (rabbit monoclonal) | (Meraldi et al., 2004) | Lab archive:  VMD5 | IF (1:1000) |
| Antibody | anti-PP1 (E-9) (mouse monoclonal) | Santa Cruz Biotechnology | sc-7482 | WB (1:5000) |
| Antibody | anti-DSN1 pSer100 (rabbit monoclonal) | [(Welburn et al. 2010)](https://paperpile.com/c/OldSzW/3hR1) | Cheeseman Lab identifier: 19.2A | IF (1:1000)  Donated by Iain Cheeseman |
| Antibody | CREST (human antisera) | Europa | FZ90C-CS1058 | IF (1:2000 or 1:1000) |
| Antibody | anti-GST (rabbit polyclonal) | Santa Cruz Biotechnology | sc-459 | WB (1: 500) |
| Recombinant DNA reagent | (pCMV-AC-GFP) SPAG5 ORF | OriGENE | RG201783 | Source of Astrin ORF for generating the plasmids of this paper. |
| Recombinant DNA reagent | (pcDNA5 FRT/TO) YFP-Astrin WT Res (plasmid) | This paper | Lab archive: VMD686 | To generate HeLa Fip-In cell lines. siRNA resisitant. |
| Recombinant DNA reagent | (pcDNA5 FRT/TO) YFP-Astrin 4A Res (plasmid) | This paper | Lab archive: VMD687 | To generate HeLa Fip-In cell lines. siRNA resistant. |
| Recombinant DNA reagent | (pcDNA5 FRT/TO) YFP-Astrin ∆70 Res (plasmid) | This paper | Lab archive: VMD688 | To generate HeLa Fip-In cell lines. siRNA resistant. |
| Recombinant DNA reagent | (pEYFP C1) YFP-Astrin WT Res (plasmid) | This paper | Lab archive: VMD672 | siRNA resistant; Can be obtained from Draviam lab or Ximbio |
| Recombinant DNA reagent | (pEYFP C1) YFP-Astrin 4A Res (plasmid) | This paper | Lab archive: VMD673 | siRNA resistant; Can be obtained from Draviam lab or Ximbio |
| Recombinant DNA reagent | (pEYFP C1) YFP-Astrin ∆70 Res (plasmid) | This paper | Lab archive: VMD674 | siRNA resistant; Can be obtained from Draviam lab or Ximbio |
| Recombinant DNA reagent | (pEGFP N1) Astrin-GFP WT (plasmid) | This paper | Lab archive: VMD636 | Can be obtained from Draviam lab or Ximbio |
| Recombinant DNA reagent | (pEGFP N1) Astrin-GFP 4A (plasmid) | This paper | Lab archive: VMD648 | Can be obtained from Draviam lab or Ximbio |
| Recombinant DNA reagent | (pEGFP N1) Astrin-GFP ∆70 (plasmid) | This paper | Lab archive: VMD643 | Can be obtained from Draviam lab or Ximbio |
| Recombinant DNA reagent | (pEGFP N1) Astrin-GFP WT Res (plasmid) | This paper | Lab archive: VMD669 | siRNA resistant; Can be obtained from Draviam lab or Ximbio |
| Recombinant DNA reagent | (pEGFP N1) Astrin-GFP 4A Res (plasmid) | This paper | Lab archive: VMD670 | siRNA resistant; Can be obtained from Draviam lab or Ximbio |
| Recombinant DNA reagent | (pEGFP N1) Astrin-GFP ∆70 Res (plasmid) | This paper | Lab archive: VMD671 | siRNA resistant; Can be obtained from Draviam lab or Ximbio |
| Recombinant DNA reagent | (pECFP N1) Astrin-CFP WT (plasmid) | This paper | Lab archive: VMD627 | Can be obtained from Draviam lab or Ximbio |
| Recombinant DNA reagent | (pECFP N1) Astrin-CFP 4A (plasmid) | This paper | Lab archive: VMD628 | Can be obtained from Draviam lab or Ximbio |
| Recombinant DNA reagent | (pEGFP C1) mCherry-GBP-PP1_γ_ (plasmid) | This paper | Lab archive: VMD619 | GFP tag of backbone swapped with mCherry. |
| Recombinant DNA reagent | (pEGFP C1) mCherry-GBP-PP1_γ_ ^F286A^ (plasmid) | This paper | Lab archive: VMD644 | GFP tag of backbone swapped with mCherry. |
| Recombinant DNA reagent | (pEGFP C1) mCherry-GBP-PP1_γ_ ^F286A D71N^ (plasmid) | This paper | Lab archive: VMD645 | GFP tag of backbone swapped with mCherry. |
| Recombinant DNA reagent | (pEYFP N1) Ndc80^HEC1^-YFP (plasmid) | This paper | Lab archive: VMD64 | Can be obtained from Draviam lab or Ximbio |
| Recombinant DNA reagent | (pECFP C1) NUF2-CFP (plasmid) | This paper | Lab archive: VMD115 | Can be obtained from Draviam lab or Ximbio |
| Recombinant DNA reagent | CenpB-Ds-RED (plasmid) | [(Shrestha and Draviam 2013)](https://paperpile.com/c/OldSzW/XvlN) | Lab archive: VMD59 | Can be obtained from Draviam lab or Ximbio |
| Recombinant DNA reagent | (pGAT3) His-GST-PP1_γ_(7-323) (plasmid) | Kindly donated by J. Peränen and M. Hyvönen | Lab archive: VMD577 | From unpublished work. To express His-GST-PP1_γ_ in BL21. |
| Recombinant DNA reagent | pGEX-6P-1 | Kindly donated by Tom Blundell | Lab archive:  VMD165 | To express GST in BL21. |
| Sequence-based reagent | Stealth negative siRNA | Invitrogen | 12,935–300 | Used as negative control |
| Sequence-based reagent | siRNA for Astrin (3’ UTR) | Sigma-Aldrich |  | GACUUGGUCUGAGACGUGAtt |
| Sequence-based reagent | siRNA for Astrin (ORF) | Sigma-Aldrich |  | UCCCGACAACUCACAGAGAAAUU  From [(Thein et al. 2007)](https://paperpile.com/c/OldSzW/5FFu) |
| Peptide, recombinant protein | His-GST-PP1_γ_(7-323) | This paper |  | Purified from *E. coli* BL21 cells. |
| Peptide, recombinant protein | GST | This paper |  | Purified from *E. coli* BL21 cells. |
| Chemical compound, drug | Oligofectamine | Thermo Fisher | 12252011 | Used for transfecting siRNA oligonucleotides into human cells. |
| Chemical compound, drug | TurboFect | Fisher | R0531 | Used for transfecting plasmid DNA into human cells. |
| Chemical compound, drug | DharmaFECT duo | Dharmacon | T-2010 | Used for transfecting plasmid DNA into human cells. |
| Chemical compound, drug | Monastrol | TOCRIS | 1305 | Used at 10 µM final concentration. |
| Chemical compound, drug | STLC | TOCRIS | 83265 | Used at 20 µM final concentration. |
| Chemical compound, drug | MG132 | TOCRIS | 1748 | Used at 10 µM final concentration. |
| Chemical compound, drug | Taxol | Sigma-Aldrich | T7191 | Used at 100 nM final concentration. |
| Chemical compound, drug | ZM447439 | TOCRIS | 2458 | Used at 10 µM final concentration. |
| Chemical compound, drug | DAPI | Sigma-Aldrich | D9542 | IF (1:2000 from a 5 ng/µl stock) |
| Chemical compound, drug | Sir-DNA kit | Spiro-Chrome | SC007 | Used at 0.25 µM final concentration. |
| Chemical compound, drug | Protease/Phosphatase Inhibitor Cocktail (100X) | Cell Signalling Technology | 5872S | Used at 1X final concentration. |
| Software, algorithm | FIJI | [(Schindelin et al. 2012)](https://paperpile.com/c/OldSzW/ZU9X) |  |  |
| Software, algorithm | SoftWoRx^TM^ | GE Healthcare |  |  |
